# Multiple Pulmonary Papillary Adenoma With Malignant Potential Accompanied by ERBIN–BRAF Gene Fusion and APC Mutation: A Case Report and Literature Review

**DOI:** 10.1155/carj/3589904

**Published:** 2026-10-09

**Authors:** Ning Ao, Hui Wu, Wenping Li, Qiwen Zhao, Zhenyang Yu, Mingang Duan, Sixin Zhang, Li Jin, Mei Liu

**Affiliations:** ^1^ Department of Pathology Sixth Medical Center of Chinese PLA General Hospital Beijing China; ^2^ Department of Radiology Sixth Medical Center of Chinese PLA General Hospital Beijing China

**Keywords:** case report, ERBIN–BRAF gene fusion, malignant potential, multiple pulmonary papillary adenoma

## Abstract

**Background:**

Pulmonary papillary adenoma is currently considered a benign tumor; however, a few cases have been reported in the past with potential for malignancy. Therefore, the histological nature of papillary adenomas remains controversial. This article presents a rare case of a multifocal papillary adenoma with uncertain malignant potential, featuring an ERBIN–BRAF gene fusion and an APC mutation, along with a literature review, to enhance understanding of this tumor.

**Case Description:**

The patient sought medical consultation due to symptoms of cough and sputum production. Imaging showed a high‐density, lobulated nodule in the posterior basal segment of the left lower lobe, surrounded by multiple smaller nodules. Postpulmonary lobectomy, histopathological examination revealed a large nodule composed of a single papillary structure, with the papillary surface covered by a single layer of cuboidal or low‐columnar epithelium. Local areas showed transition to surrounding alveolar epithelium, and well‐formed glands were observed in the proliferative fibrous tissue, with local stromal fibrosis. Some tumor cells in the surrounding small nodules grew in a lepidic fashion, with cellular morphology similar to that of the large nodule. Immunohistochemical staining showed that tumor cells were positive for TTF‐1, CK, CK7, and Napsin A, with a few cells expressing P53 and a Ki‐67 index of approximately 8%. Next‐generation sequencing (NGS) identified an APC gene mutation (Exon 16, p.E1306∗) and an ERBIN exon3–BRAF exon11 fusion. Given that BRAF alterations have been implicated in lung carcinogenesis, a similar BRAF‐mediated tumorigenic pathway may be active in this tumor; therefore, complete resection of the adenoma may be indicated. The tumor’s multifocal distribution and molecular alterations suggest that this case represents a papillary adenoma with uncertain malignant potential.

**Conclusions:**

Currently, there is no established standard treatment for such tumors, and the tumor in this case underwent pulmonary lobectomy. Future studies should accumulate more cases to clarify the prognosis and treatment guidelines for this type of tumor.

## 1. Introduction

Pulmonary papillary adenoma is a rare benign tumor that predominantly occurs in the peripheral lung. In 1980, Spencer et al. first described 2 cases of peripheral pulmonary papillary adenoma, noting that the morphology of the tumor cells resembles that of Clara cells; in one case, a portion of the tumor cells protruded into the surrounding alveolar space and capsule, suggesting possible invasion. To date, there have been 39 reported cases of pulmonary papillary adenoma in the English literature (see Tables 1 and 2), with 8 cases indicating a certain malignant potential. These cases exhibited varying atypia, increased nuclear–cytoplasmic ratios, nuclear crowding or overlapping, and a glandular‐like arrangement similar to in situ adenocarcinoma. Some tumor cells invaded the surrounding alveolar septa, small venules, surrounding capsules, and subpleural lymphatics, disseminating along the peripheral bronchial lumen [1–21]. Currently, clinical treatment methods generally include wedge resection, segmentectomy, and lobectomy. Until now, only one case of multifocal disease has been reported; in this case, the tumor was not excised and has not shown progression over a 6‐year follow‐up period, though it was not subjected to genetic testing. More similar cases warrant further investigation and reporting.

**TABLE 1:** Summary of previous reports and present case.

Case	Author (year)	Age (years)/sex	Respiratory symptoms	CT chest performance (diameter)	Surgery	Intraoperative rapid diagnosis	Macro	Capsule
1	Spencer et al. (1980) [1]	26/F	Cough, pyrexia, and slight hemoptysis, wheezing, respiration, chest pain	Mass (4 cm)	Lobectomy	ND	NM	−
2	Spencer et al. (1980) [1]	7/M	−	Opacity	Lobectomy	ND	NM	+
3	Fantone et al. (1982) [2]	25/F	−	Mass (2.5 cm)	Lobectomy	ND	Soft, brown, granular	−
4	Noguchiet al. (1986) [3]	57/M	−	Mass (1.5 cm)	Lobectomy	ND	Ivory yellow, granular	−
5	Hegg et al. (1992) [4]	60/M	−	Mass (2.4 cm)	Lobectomy	ND	Spongy, granular, without necrosis or hemorrhage	−
6	Hegg et al. (1992) [4]	52/F	−	Mass (1.2 cm)	Lobectomy	ND	Same as above	−
7	Fukuda et al. (1992) [5]	23/M	−	Nodule (1.8 cm)	Lobectomy	ND	Hemorrhagic	−
8	Fukuda et al. (1992) [5]	56/M	−	Nodule (1.8 cm)	Wedge resection	ND	White, granular	−
9	Kurotaki et al. (1993) [6]	13/M	−	Multiple nodules (diameter from 0.5 to 2 mm)	ND	ND	Grayish white	−
10	Yamamoto et al. (1993) [7]	26/M	−	Nodule (2.0 cm)	Wedge resection	ND	Yellowish white	+
11	Sanchez et al. (1994) [8]	2months/F	−	Nodule (2.0 cm)	Wedge resection	ND	White	−
12	Mori et al. (1996) [9]	35/M	−	Nodule (2.0 cm)	Lobectomy	Imprint cytology	Soft, grayish white, partially hemorrhagic	−
13	Inada et al. (1999) [10]	51/F	−	Nodule (8 mm)	Excisional biopsy	ND	NM	−
14	Dessy et al. (2000) [11]	15/M	−	Nodule (2.5 cm)	Wedge resection	ND	Firm, grayish white, partially hemorrhagic	+
15	Dessy et al. (2000) [11]	27/M	−	Nodule (2.4 cm)	Thoracotomy	Partially encapsulated papillary tumor	Firm, grayish white	+
16	Neusuesset al. (2002) [12]	9/M	NM	Nodule (4 mm)	ND	NM	NM	−
17	Minami et al. (2004) [13]	50/M	−	Mass (25 mm)	Segmentectomy	Papillary adenoma	White, granular, and solid	−
18	Nakano et al. (2011) [14]	52/M	−	Nodule (18 mm)	Lobectomy	Adenocarcinoma	Soft, dark reddish, granular	−
19	Masunagaet al. (2012) [15]	16/F	−	Mass (30 mm)	Lobectomy	ND	Soft, spongy, and whitish	NM
20	Cornejo et al. (2013) [16]	24/M	Asthma	Mass (6 cm)	Enucleation	ND	White‐tan and yellow granular and hemorrhagic parenchyma	−
21	Lin et al. (2015) [17]	17/F	−	Mass (3.13 cm)	Wedge resection	ND	Firm and gray‐white or gray‐yellow	+
22	Frey et al. (2016) [18]	78/F	−	Mass (3.9 cm)	ND	ND	NM	NM
23	Hu et al. (2020) [19]	65/M	−	Mass (4.2 cm)	ND	ND	NM	NM
24–38	Zhou et al. (2020) [20] 15 cases	The average age at disease onset was 61.3 years (6 M, 9 W).	The clinical symptoms are unspecific, such as cough, sputum, congestive heart failure, and chest pain	Some revealed a mass or nodule. Some showed pneumonia, bronchiectasis, ground‐glass opacity, and interstitial fibrosis under the pleura	Five patients, including two with malignant potential, underwent lobectomy	ND	Grayish brown and soft	−
39	Liu et al. (2022) [21]	66/F	Dry cough	Mass (5.5 cm)	Thoracoscopy	Papillary adenoma	Gray‐white, solid, and granular	−
40	Present case	66/M	Cough	Mass (4.3 cm)	Lobectomy	NM	Gray‐white, solid	+

*Note:* −, negative; +, positive.

Abbreviations: ND, not done; NM, not mentioned.

**TABLE 2:** Summary of previous reports and present case.

Case	Author (Year)	Morphological dysplasia	Invasion	Immunohistochemistry	Molecular detection	Type	Outcome
1	Spencer et al. (1980) [1]	−	−	ND	ND	Benign	ND
2	Spencer et al. (1980) [1]	−	Adjacent alveoli	ND	ND	Malignant potential	ND
3	Fantone et al. (1982) [2]	−	−	No IR for SP‐A and Clara cell protein	ND	Benign	ND
4	Noguchiet al. (1986) [3]	−	−	IR for SP‐A	ND	Benign	NED 8 years
5	Hegg et al. (1992) [4]	−	−	No IR for SP‐A nor for Clara cell protein	ND	Benign	NED 2 years
6	Hegg et al. (1992) [4]	−	−	IR for Clara cell protein. No IR for SP‐A	ND	Benign	NED 11 months
7	Fukuda et al. (1992) [5]	−	−	ND	ND	Benign	NED 10 years
8	Fukuda et al. (1992) [5]	−	−	ND	ND	Benign	NED 2 years
9	Kurotaki et al. (1993) [6]	−	−	IR for SP‐A (solid only weakly positive), EMA, AE1/AE3	ND	Benign	NED 6 years
10	Yamamoto et al. (1993) [7]	−	−	IR for SP‐A	DNA ploidy	Benign	NED 2 years
11	Sanchez et al. (1994) [8]	−	−	ND	ND	Benign	NED 2 years
12	Mori et al. (1996) [9]	−	Adjacent alveoli, small venules	IR for SP‐A, SP‐B, cytochrome P‐450, CEA	ND	Malignant potential	NED 3 years
13	Inada et al. (1999) [10]	−	−	IR for SP‐A, CK	ND	Benign	NED 4 months
14	Dessy et al. (2000) [11]	−	Capsule	IR for SP‐A, AE1/AE3, CEA, TTF‐1, Ki‐67 (less than 2%)	ND	Malignant potential	NED 9 years
15	Dessy et al. (2000) [11]	−	Capsule, adjacent alveoli, subpleural lymphatics	IR for SP‐A, AE1/AE3, CEA, TTF‐1, Ki‐67 (less than 2%)	ND	Malignant potential	NED 2 years
16	Neusuess et al. (2002) [12]		−	IR for CK and NSE (focally weak expression)	ND	Benign	NED 6 months
17	Minami et al. (2004) [13]	The nuclei of the tumor cells were uniform in size and atypical	−	IR for SP‐A and CEA, PCNA (slightly positive)	ND	Benign	NED 35 months
18	Nakano et al. (2011) [14]	Solid components presented more nuclear atypia of tumor cells than the papillary and septal components. Tumor cells showed irregular nuclear size, and some nuclei contained a clear eosinophilic nucleolus	−	IR for TTF‐1 and SP‐A (positive in solid components) and UP‐1 (slightly positive in the papillary and septal components). P53 and Ki67 (more positive reaction in the solid components)	No mutation was detected in the EGFR gene and the KRAS gene.	Benign	NED 38 months
19	Masunaga et al. (2012) [15]	Tumor cells grew in a lepidic fashion (at the periphery)	−	IR for FGFR2	FGFR2IIIb and KGF mRNA were detected in the tumor cells	Benign	NM
20	Cornejo et al. (2013) [16]	Tumor cells proliferated along the alveolar walls in a lepidic manner	−	IR for TTF‐1 pancytokeratin (CK), CK7, epithelial membrane antigen, and c‐Myc	No mutation was detected in the EGFR, KRAS, and BRAF genes	Benign	NED 6 months
21	Lin et al. (2015) [17]	−	−	IR for TTF‐1, EMA, CK, CK7, SPA, P63, and Napsin A	ND	Benign	NED 12 months
22	Frey et al. (2016) [18]	−	−	IR for TTF‐1 and Napsin A	ND	Benign	NED 26 months
23	Hu et al. (2020) [19]	Some tumor cells had more irregular nuclear membranes and crowded or overlapping larger nuclei with moderate cytologic atypia. Some atypical cells were arranged in a glandular pattern	Fibrous stroma	IR for TTF1, Napsin A, and CK7	ND	Malignant potential	ND
24–38	Zhou et al. (2020) 20 15 cases	Two cases had different sizes and a disorderly and uneven arrangement. The nuclei were strongly stained with HE, and mitotic figures were easy to see	Some tumor cells had infiltrated between the interstitial regions	IR for CK7, Vim, CK20, and TTF1. Special stains for PAS and AB	ND	Two cases were malignant potential	One patient dead, remaining patients experienced a good outcome
39	Liu et al. (2022) [21]	Tumor cells in some areas were crowded and arranged pseudostratified with obvious nucleoli	Tumor locally protruded into the surrounding lung tissue	IR for Napsin A, TTF‐1, CK7, and P53 (weakly staining). The Ki‐67 index was about 6%, and close to 20% in hot spots	ND	Malignant potential	NED 2 years
40	Present case	Tumor cells grew in a lepidic fashion (at the periphery)	−	IR for TTF‐1, CK, CK7, and Napsin A. The Ki‐67 index was about 8%	ERBIN–BRAF gene fusion and APC mutation	Malignant potential	NED 2 years

*Note:* −, negative; NED, no evidence of disease; SP‐A, surfactant apoprotein A.

Abbreviations: ND, not done; PCNA, proliferating cell nuclear antigen.

Regarding the origin of pulmonary papillary adenoma, we analyzed several early case reports. In 1982, Fantone et al. published a report of a pulmonary papillary adenoma, where electron microscopy revealed that the tumor cells contained lamellar bodies resembling Type II alveolar epithelial cells and dense granules found in Clara cells, indicating that the tumor likely arises from both cell types (Type II alveolar epithelial cells and Clara cells) [2]. In 1986, Noguchi et al. again utilized electron microscopy to suggest that the tumor may originate from “stem cells” within the bronchiolar–alveolar epithelium, with the disappearance of elastic fibers in the stroma during cell growth, which contrasts with the growth pattern of bronchial–alveolar cell carcinoma. Additionally, some researchers propose that pulmonary papillary adenoma exhibits a growth pattern similar to that of sclerosing alveolar cell tumors [3]. In 1993, Kurotaki et al. reported the first case of multifocal pulmonary papillary adenoma, further supporting the notion that it may represent an early manifestation of sclerosing alveolar cell tumors [6].

This study reports a case of multifocal pulmonary papillary adenoma, analyzing its clinical and pathological features along with molecular alterations, and compares these findings with all previously reported cases of pulmonary papillary adenoma to further determine the nature of this tumor.

## 2. Materials and Methods

### 2.1. Reagents

Cytokeratin 7 (CK7) (clone number: UMAB161) and Ki67 (clone number: UMAB107) were purchased from Beijing Zhongshan Golden Bridge Biotechnology Co., Ltd. CK5/6 (clone number: MX040), P40 (clone number: MXR010), P53 (clone number: MX008), thyroid transcription factor 1 (TTF‐1) (clone number: 8G7G3/1), Napsin A (aspartic proteinase A) (clone number: MX015), Chromogranin A (CgA) (clone number: RMA‐0548), synaptophysin (Syn) (clone number: MAB‐0742), and CD56 (neural cell adhesion molecule) (clone number: MX039) were obtained from Fuzhou Maixin Bio‐Tech Development Co., Ltd. The EnVision FLEX/HRP reagent and ethylenediaminetetraacetic acid (EDTA) antigen retrieval solution were purchased from DAKO in Denmark. The AB‐PAS and elastic fiber staining kits were obtained from Anhui Regend Biotechnology Co., Ltd.

### 2.2. EnVision Method for Immunohistochemical Staining

Surgical specimens were fixed in 4% neutral formaldehyde solution and prepared according to standardized protocols. Following dehydration and paraffin embedding, the specimens were sectioned into 3‐ to 4‐μm‐thick slices. The paraffin sections were placed in an oven at 80°C for 20 min and then cooled to room temperature. They were deparaffinized in Xylene I and II for 10 min each, followed by hydration through gradient ethanol. The sections were washed three times for 3 min each with phosphate‐buffered saline (PBS) and underwent high‐temperature, high‐pressure antigen retrieval in pH 6.0 EDTA solution for 2 min. After three additional washes with PBS for 3 min each, the subsequent procedures were carried out using the DAKO fully automated immunohistochemistry instrument (Autostainer Link 48): incubation with 3% hydrogen peroxide for 10 min, followed by rinsing three times with PBS for 1 min each. The primary antibody was then incubated at room temperature for 1 h, followed by three rinses with PBS for 1 min each. The EnVision FLEX/HRP reagent was incubated at room temperature for 20 min, with subsequent washing three times with PBS for 1 min each. The color was developed with DAB at room temperature for 10 min. The sections were counterstained with hematoxylin, dehydrated through a gradient of ethanol, and mounted with neutral resin.

### 2.3. Extraction of DNA and RNA and Library Preparation

Tissue samples that were fixed in formalin and embedded in paraffin were processed using the MagPure FFPE DNA LQ Kit (Catalog No. D6323, Magen) and the AmoyDx FFPE RNA Extraction Kit (Catalog No. 8.02.24101X036G, Aide) to extract total DNA and RNA, respectively. The DNA samples were first fragmented into 200‐ to 250‐bp pieces using an ultrasonic disruptor (Covaris LE220) and then constructed into next‐generation sequencing (NGS) libraries using the Pan‐Cancer Tissue Sample (Master Panel) Gene Detection Kit (Catalog No. 8.06.0130, Aide) through steps involving end repair, A‐tailing, adapter ligation, and PCR amplification. Based on the DV200 values assessed by the Agilent 2100 Bioanalyzer, RNA samples were incubated at 95°C for 0–15 min for fragmentation. The fragmented RNA was processed using the Pan‐Cancer Tissue Sample (Master Panel) Gene Detection Kit, followed by reverse transcription, cDNA synthesis, and strand‐specific library preparation to construct sequencing libraries.

### 2.4. Hybrid Capture and Sequencing

DNA and RNA libraries were captured using the Pan‐Cancer Tissue Sample (Master Panel) Gene Detection Kit (Catalog No. 8.06.0130, Aide). The captured products were then amplified and quantified using the Quantus Fluorometer, followed by library fragment assessment with the Agilent 2100 Bioanalyzer. After merging the libraries, sequencing was performed on the Illumina NovaSeq 6000 platform (Illumina) using a paired‐end 150‐bp read strategy. The sequencing data were analyzed and annotated using an internally developed pipeline.

### 2.5. Statistical Analysis

This is a single‐case report, and no statistical calculations of any kind were carried out throughout the manuscript. No *P*‐values are presented. The literature review is purely narrative without quantitative statistical synthesis; therefore, no statistical software was utilized. SAMPL guidelines were followed where relevant. No artificial intelligence tools were used in the research methodology, data collection, data analysis, or literature‐review processes.

## 3. Case Presentation

The patient is a 66‐year‐old male who presented with a cough and expectoration of white mucoid sputum of approximately 1‐year duration without an identifiable infectious or other cause, and no specific medical treatment was administered before or after surgery. He had no history of pneumonia, pleural effusion, or thoracic surgery, chronic obstructive pulmonary disease (COPD), or other underlying respiratory conditions. The patient was a smoker with a smoking history of more than 10 years. He worked as a company manager, with no history of silicosis or occupational dust exposure. The patient’s father had a history of a lung tumor, although the exact pathological type is unknown; no other family members had a history of pulmonary disease or malignancy. Sputum microbiological or cytological examination was not performed prior to surgery. A chest computed tomography (CT) scan revealed a high‐density, lobulated mass in the posterior basal segment of the left lower lobe, measuring approximately 40 mm in diameter (Figure 1A–C). Multiple small nodules, measuring about 1–4 mm in diameter, were observed surrounding the mass, some of which were adjacent to the pleura (Figure 1D). The mass had clear borders and was distributed along the left pulmonary artery tree in the posterior segment, while the surrounding small nodules followed the distribution of the vascular bronchial bundle (Figure 2). Based on these imaging features, the clinical suspicion for a malignant pulmonary neoplasm was high. Serum tumor markers, including carcinoembryonic antigen (CEA), cytokeratin 19 fragment (CYFRA21‐1), neuron‐specific enolase (NSE), and cancer antigen 19‐9 (Ca19‐9), were all within normal ranges. Serum infection markers, including C‐reactive protein (CRP), were also within normal limits.

**FIGURE 1:**
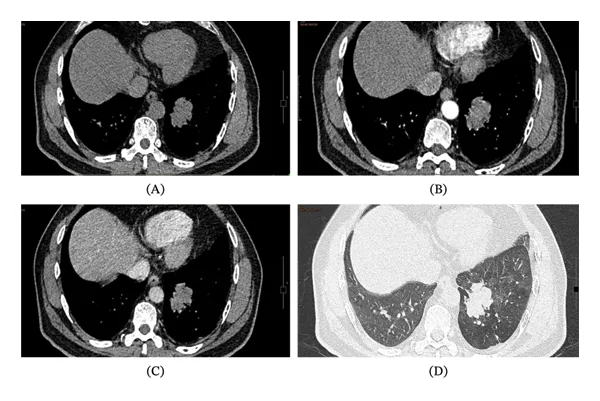
Chest computed tomography (CT) findings. (A) Plain scan: an irregular, lobulated mass near the vascular bundle in the basal segment of the left lower lobe, with a CT value of approximately 40 Hounsfield units (HU). (B) Arterial phase of contrast‐enhanced CT: The mass showed slight enhancement, with a CT value of approximately 78 HU. (C) Venous phase: CT value of approximately 63 HU. The lesion measured 4.8 × 3.5 × 2.5 cm; the bronchus‐adjacent portion was slightly pushed and displaced, and the branch bronchus of the anterior inner basal segment passed through the lesion without obvious truncation. (D) Multiple solid nodules (1–4 mm) were observed near the distal end of the lesion. Abbreviations: CT, computed tomography; HU, Hounsfield unit.

**FIGURE 2:**
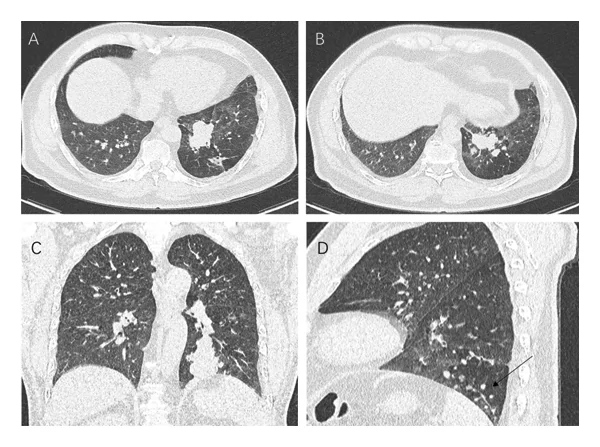
(A) Axial view: multiple solid nodules near the distal end of the lesion. (B) Coronal view: the consolidation was distributed along the bronchial tree of the posterior segment of the left lung (upper image). (C, D) Sagittal view: some nodules were distributed along the vascular bronchial bundle (long arrow) (lower image).

Clinical thoracoscopic‐assisted left lower lobectomy and mediastinal lymph node dissection were performed, and fresh tissue was sent for intraoperative frozen pathological diagnosis. The specimen from the left lower lobe revealed a large lobulated nodule measuring 4.8 × 3.5 × 2.5 cm, with a grayish‐white cut surface that was firm and homogeneous. Surrounding the large nodule, multiple small nodules measuring 0.1–0.4 cm in maximum diameter were observed, with some adjacent to the visceral pleura but not involving the bronchial wall. Paraffin‐embedded sections showed that the surface of the large nodule was encapsulated by fibrous tissue, with tumor cells arranged in a papillary or glandular pattern, local interstitial fibrous tissue proliferated (Figures 3A,B). Papillary or glandular structures were lined by a single layer of cuboidal or low‐columnar epithelium, with uniform epithelial cell morphology characterized by round or oval nuclei located basally, and cytoplasm that was either clear or eosinophilic. In some areas, the cell arrangement was disordered, with transition to the surrounding alveolar epithelium, some of which locally protruded into the surrounding lung tissue, where pale pink secretions were observed (Figures 3C,D). Within the surrounding small nodules, some cells grew in a lepidic fashion, with cellular morphology similar to that of the large nodule (Figure 4). The tumor cells showed no atypia, and there were no signs of necrosis or mitotic figures. Immunohistochemical analysis indicated that tumor cells expressed TTF‐1, CK, CK7, and Napsin A, while p40 and CK5/6 were negative; a small number of cells expressed P53, and the Ki67 proliferation index was approximately 8% (Figure 5). Syn, CgA, and CD56 were negative. NGS revealed an adenomatous polyposis coli (APC) gene mutation (Exon 16, p.E1306∗) and ERBIN Exon3–BRAF Exon11 fusion. Based on the morphological features, immunohistochemical profile, and molecular findings, we diagnosed this case as multifocal papillary adenoma with indeterminate malignant potential.

**FIGURE 3:**
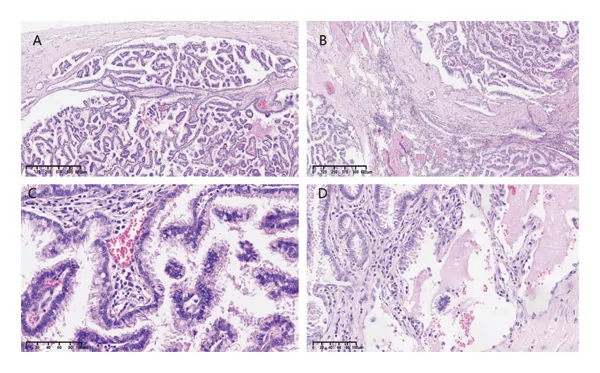
Histopathological findings (hematoxylin–eosin [HE] staining). (A, B) The large nodule was encapsulated by fibrous tissue, with local interstitial fibrous tissue proliferation inside (magnification × 40). (C) Tumor cells showed papillary growth, with basally located nuclei and clear or eosinophilic cytoplasm (magnification × 200). (D) Tumor cells transitioned into the surrounding alveolar epithelium, with some cells protruding into the alveolar space (magnification × 200).

**FIGURE 4:**
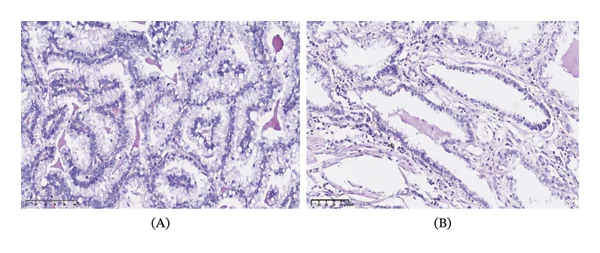
(A) Cellular morphology of the surrounding small nodules resembled that of the large nodule (magnification × 200). (B) Some cells grew in a lepidic fashion along the alveolar walls (magnification × 200).

**FIGURE 5:**
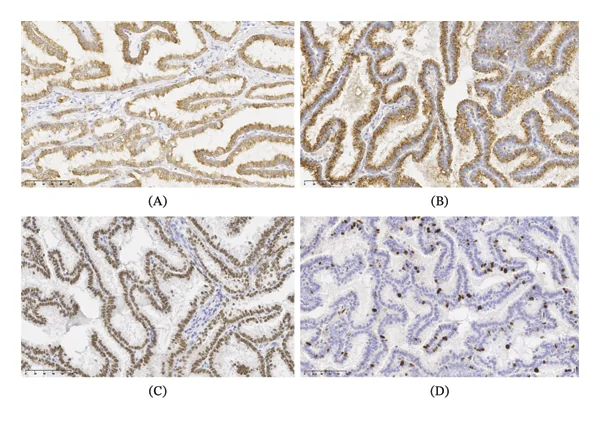
Immunohistochemical staining (magnification × 100). Tumor cells showed diffuse positive expression of CK7 (A), Napsin A (B), and TTF‐1 (C); the Ki‐67 proliferation index was approximately 8% (D). Abbreviations: CK, cytokeratin; TTF‐1, thyroid transcription factor‐1. All figures were derived from original clinical data and were not generated or edited by artificial intelligence tools.

## 4. Discussion

Pulmonary papillary adenoma is more commonly found in males, with an onset age ranging from 2 months to 78 years (median, 26.5 years; calculated from the 24 previously reported cases with individual‐level age data). The clinical manifestations are nonspecific, and patients are often diagnosed through routine examinations. In a literature review of 39 reported cases, most CT scans revealed solitary nodules or masses in the lung periphery with diameters ranging from 1 to 6 cm, characterized by smooth, rounded, or oval edges. Occasionally, nodules or masses were observed in the central lung regions (hilar area and main bronchi), with only 5 cases presenting as pneumonia, 3 as bronchiectasis, 1 as ground‐glass opacities, and 1 as subpleural interstitial fibrosis (Table 1). To date, only one case of multifocal pulmonary papillary adenoma has been reported by Kurotaki, involving a 13‐year‐old male patient with no clinical symptoms, whose X‐ray showed diffuse reticular small nodules in both lungs, initially suspected to be pulmonary fibrosis [6]. In this case, CT imaging revealed a lobulated mass in the posterior basal segment of the left lower lobe, along with multiple small nodules surrounding it, making the imaging features particularly distinctive.

The diagnosis in this case was challenging due to the tumor exhibiting both benign characteristics and evidence of malignancy. First, the features supporting a benign tumor include a fibrous capsule surrounding the large nodule, clear borders, and a well‐defined papillary or glandular arrangement covered by a single layer of cuboidal or low‐columnar epithelium. The epithelial cells were relatively uniform in shape, with round or oval nuclei located basally, clear or eosinophilic cytoplasm, and no mitotic figures present. The epithelial cells expressed TTF‐1, CK, CK7, and Napsin A, consistent with the descriptions of benign pulmonary papillary adenomas reported in the literature. One reported case of benign lesions showed fibrous encapsulation [7], and under low magnification, the tumor displayed papillary and glandular arrangements, as well as cuboidal or columnar epithelial cells. Under high magnification, the cell nuclei were round or oval, lightly stained, and showed no mitosis or other abnormalities. CKs, TTF‐1, surfactant proteins, p63, epithelial membrane antigen, and Napsin A are expressed in tumor epithelial cells but never in stromal cells. On the other hand, the evidence supporting malignant behavior includes the imaging characteristics of multiple nodules and suspected airway dissemination, which is reported for the first time in this case. Liu reported a case of a pulmonary papillary adenoma with indeterminate malignant potential, where the tumor cells exhibited active proliferation, with Ki‐67 proliferation indices in hotspot areas reaching 20% [21]. In this case, the tumor cells showed a Ki‐67 proliferation index of 8%, also suggesting potential malignant behavior. In summary, this case was diagnosed as a pulmonary papillary adenoma with indeterminate malignant potential.

In this case, we detected an APC (E1306∗) mutation for the first time in a patient with multifocal pulmonary papillary adenoma. It is known that familial adenomatous polyposis (FAP) results from germline mutations in the APC gene located on chromosome 5q. Patients with FAP typically develop hundreds of colonic adenomatous polyps beginning in adolescence, as the activation of the APC gene initiates a series of “adenoma–carcinoma” molecular pathways that ultimately lead to colorectal cancer [22]. Naoyuki Iwahashi et al. collected plasma samples from patients with colorectal cancer metastasized to the ovaries, as well as paraffin‐embedded ovarian tumor samples, and found mutations in the APC (E1306∗), KRAS G13D, and TP53 H193Y genes [23]. The presence of the APC gene mutation in this tumor suggests that similar “adenoma–carcinoma”‐associated molecular pathways may also be activated in the lungs, indicating a potential for malignant transformation. Therefore, more related case reports and further research are needed in the future.

This case represents the first discovery of a BRAF Exon 11 and ERBIN gene Exon 3 fusion in pulmonary papillary adenoma. BRAF is a member of the RAF kinase family and plays a crucial role in the mitogen‐activated protein kinase (MAPK) signaling pathway. BRAF gene fusions were first reported in thyroid cancer in 2005, where an in‐frame fusion between AKAP9 gene Exons 1–8 and BRAF Exons 9–18 (including the kinase domain) resulted in the loss of the self‐inhibitory N‐terminal region of the BRAF protein, leading to the activation of intracellular effector molecules and enhanced kinase activity via the MAPK pathway [24]. Recently, the Memorial Sloan Kettering Cancer Center (MSKCC) conducted NGS on 10,000 patients with advanced cancer and identified approximately 39 cases of BRAF fusions (with non–small cell lung carcinoma accounting for about 0.38%). Thus, BRAF fusion genes may serve as oncogenic drivers and represent potential targets for targeted therapy [25]. The presence of a BRAF mutation in this case suggests the potential activation of MAPK pathway‐related factors, providing significant insights into the genetic variations associated with this type of tumor.

The differential diagnosis for this case includes sclerosing pneumocytoma and pulmonary papillary adenoma, among others. After analyzing and comparing the similarities and differences between pulmonary papillary adenoma and sclerosing pneumocytoma, we found the following: First, sclerosing pneumocytoma and papillary adenoma exhibit similar imaging characteristics, presenting as well‐defined coin‐like nodules. Second, the structural characteristics of the papillary growth pattern in sclerosing pneumocytoma, including cytological morphology, ultrastructure, and immunohistochemical studies, also emphasize its similarity to pulmonary papillary adenoma. This suggests that the epithelial cell origin of both tumors is the same, as both are derived from Type II alveolar epithelial cells and Clara cells. Third, although literature reports indicate that the growth patterns of pulmonary papillary adenoma resemble those of sclerosing pneumocytoma—for example, Nakano et al. reported a case of pulmonary papillary adenoma with focal solid growth [14], and Cornejo et al. identified a case with prominent sclerotic features based on morphological characteristics [16]—most pulmonary papillary adenomas do not have solid or sclerotic regions. Fourth, under microscopic examination, sclerosing pneumocytoma shows two cell types: cuboidal surface cells and round stromal cells [6]. Immunohistochemically, the surface cells express AE1/AE3 and CK7, while the stromal cells express vimentin; both cell types express TTF‐1 and EMA. In contrast, pulmonary papillary adenoma shows only one type of cell under microscopic examination. Fifth, molecular detection in sclerosing pneumocytoma reveals AKT1 mutations, with some cases showing BRAFV600E mutations, which differs from pulmonary papillary adenoma.

We further analyzed the similarities and differences between pulmonary papillary adenoma and alveolar adenoma and found the following: First, both exhibit similar imaging characteristics, often presenting as solitary nodules [26]. Second, alveolar adenoma is characterized by multiple cystic cavities lined with Type II alveolar epithelial cells of varying thickness. The cysts may contain eosinophilic proteinaceous fluid, and the stroma may show varying numbers of spindle cells, but no papillary structures are observed [27]. Third, the lining of the cystic cavities in alveolar adenoma is composed of cuboidal epithelial cells that immunohistochemically express CK and TTF‐1, while the spindle cells in the stroma express SMA, MSA, and CD34. In contrast, pulmonary papillary adenoma shows only one type of cell under microscopic examination. Fourth, to date, no specific genetic mutations have been reported in alveolar adenoma.

Regarding the prognosis and treatment of this type of tumor, literature reports describe eight cases of pulmonary papillary adenoma with certain malignant potential that underwent wedge resection or lobectomy after diagnosis. All but one patient, who died following lobectomy, had favorable postoperative follow‐up results without recurrence. Therefore, there is currently no established surgical standard for the treatment of these tumors. However, due to their potential for malignancy, it is advisable to perform radical surgeries such as segmentectomy or lobectomy following diagnosis. In this case, the patient presented imaging characteristics suggestive of a malignant lung tumor, leading the surgeons to perform a left lower lobectomy. The patient has remained in good overall condition during the 2‐year follow‐up period; however, small scattered nodules measuring 3–4 mm have been detected in the contralateral lung at the most recent follow‐up, and their nature requires continued radiological surveillance. Future treatment approaches for such tumors require the accumulation of more cases to further clarify their management.

## 5. Conclusion

In summary, we report for the first time a case of a very rare multifocal pulmonary papillary adenoma with indeterminate malignant potential, associated with ERBIN–BRAF gene fusion and APC mutation. Most pulmonary papillary adenomas have a good prognosis; therefore, surgical resection should be the primary treatment for these tumors. We hypothesize that some atypical papillary adenoma cases may harbor genetic mutations that could lead to malignant transformation, and clinicians should maintain a heightened level of vigilance. Future studies should aim to accumulate more cases to further elucidate the nature and prognosis of these tumors.

## Author Contributions

All authors contributed to the study conception and design. Material preparation and data collection and analysis were performed by Ning Ao, Hui Wu, Wenping Li, Qiwen Zhao, Zhenyang Yu, Mingang Duan, Sixin Zhang, Li Jin, and Mei Liu. The first draft of the manuscript was written by Ning Ao and Mei Liu, and all authors commented on previous versions of the manuscript. Mei Liu, the corresponding author, had full access to all of the data in this study and takes complete responsibility for the integrity of the data and the accuracy of the data analysis. The corresponding author (Mei Liu) affirms that this manuscript is an honest, accurate, and transparent account of the study being reported; that no important aspects of the study have been omitted; and that any discrepancies from the study as planned have been explained.

## Funding

This study did not receive any specific grant from funding agencies in the public, commercial, or not‐for‐profit sectors. The next‐generation sequencing (NGS) analysis was paid for by the patient out‐of‐pocket, and the other diagnostic procedures, including immunohistochemical staining, were performed as part of routine clinical care.

## Disclosure

All authors read and approved the final manuscript. No funding sources had any role in the study design; collection, analysis, and interpretation of data; writing of the report; or the decision to submit the report for publication.

## Ethics Statement

All procedures performed in studies involving human participants were in accordance with the ethical standards of the institutional and/or national research committee and with the 1964 Helsinki declaration and its later amendments or comparable ethical standards. According to the institutional policy of the Sixth Medical Center of Chinese PLA General Hospital, formal Institutional Review Board (IRB) ethics approval is not required for retrospective case reports involving up to two patients; written documentation of this policy can be provided upon request. Written informed consent was obtained from the patient for the publication of this case report.

## Conflicts of Interest

The authors declare no conflicts of interest.

## Data Availability

The datasets used and analyzed during the current study are available from the corresponding author upon reasonable request.
